# Discharge of a Tooth from the Ear

**Published:** 1848-01

**Authors:** Mervin Coates


*Discharge of a Tooth from the Ear.* By Mervin Coates, M. D.—The following curious case happened in my practice. At the time of its occurrence I resided in the Isle of Wight. In the summer of

1846, being myself absent from home, a friend was called upon to attend an old, poor man, who had suffered for some days from severe pain over the whole of one side of the face and head, but more intensely still about the ear. He found him feverish, in great pain, and incapable of opening his mouth; the pinna and skin lining the external meatus were highly inflamed and swollen. Warm fomentations, poultices, and purgatives, were ordered. Two days afterwards I paid him a visit. He was then in great pain, and, otherwise much in the same state as I have already described, but, in addition, there was an oozing of pus from the meatus, and almost entire closure of that passage by a whitish substance, which the patient conjectured to be a piece of onion, introduced there by the recommendation of some old woman, but which a probe detected to be bony. The patient declining to have this removed, he was recommended to continue to foment and poultice. That same night a fit of sneezing forced out the piece of bone felt by the probe, which proved to be one of the wisdom-teeth of the upper jaw; after that the man got well.—*London Lancet.*

